# A kinematic analysis of a haptic handheld stylus in a virtual environment: a study in healthy subjects

**DOI:** 10.1186/1743-0003-4-13

**Published:** 2007-05-09

**Authors:** Jurgen Broeren, Katharina S Sunnerhagen, Martin Rydmark

**Affiliations:** 1 Rehabilitation medicine, Institute of Neuroscience and Physiology, The Sahlgrenska Academy at Göteborg University, Guldhedsgatan 19, Göteborg, Sweden; 2 Mednet – Medical Informatics & Computer Assisted Education, Institute of Biomedicine, The Sahlgrenska Academy at Göteborg University, Box 420 Göteborg, Sweden

## Abstract

**Background:**

Virtual Reality provides new options for conducting motor assessment and training within computer-generated 3 dimensional environments. To date very little has been reported about normal performance in virtual environments. The objective of this study was to evaluate the test-retest reliability of a clinical procedure measuring trajectories with a haptic handheld stylus in a virtual environment and to establish normative data in healthy subjects using this haptic device.

**Methods:**

Fifty-eight normal subjects; aged from 20 to 69, performed 3 dimensional hand movements in a virtual environment using a haptic device on three occasions within one week. Test-retest stability and standardized normative data were obtained for all subjects.

**Results:**

No difference was found between test and retest. The limits of agreement revealed that changes in an individual's performance could not be detected. There was a training effect between the first test occasion and the third test occasion. Normative data are presented.

**Conclusion:**

A new test was developed for recording the kinematics of the handheld haptic stylus in a virtual environment. The normative data will be used for purposes of comparison in future assessments, such as before and after training of persons with neurological deficits.

## Background

Virtual Reality (VR) technology provides new options for conducting motor assessment and training within computer-generated 3 dimensional (3D) environments for persons with stroke and other diagnoses with motor deficits such as cerebral palsy, parkinson's disease or multiple sclerosis [1-6]. Findings in many studies suggest that training in a virtual environment has effects and indicate improvements in functional abilities. The advantages of VR are its possibility to provide both a systematic training arena and an assessment tool [7]. The potential of VR to identify the underlying deficit can facilitate the planning of clinically relevant intervention programmes targeted at a specific deficit. In addition, the accuracy of the computerized assessment can be used to measure progress objectively and to isolate more subtle aspects in patients with neurological diseases [8]. Evaluating the effect of an intervention where semi-subjective evaluations of current approaches cannot discriminate changes could be a key factor in outcome measures for rehabilitation. Most studies use matched controls to compare the performance with patients or to identify characteristics of the intervention used. The findings allow us to decide whether the results from patients are due to the impairment or if they are poorer/better then the matched controls.

In a recent study by Viau [9], a VR task was validated as a tool for studying arm movements in healthy and stroke subjects by comparing movement kinematics in a virtual environment and in the physical world. They concluded that both healthy and stroke subjects used similar movement strategies. However, the differences in movements made by healthy subjects in the two environments could be explained by the absence of haptic feedback and the use of a 2 dimensional environment instead of 3D virtual environment [9]. Bardorfer and colleagues [10] conducted a study in patients with neurological diseases for hand motion analysis using the PHANTOM Premium 1.5-haptic interface (rendering sensory feedback). They evaluated a test for kinematic analysis to measure motor abilities. Since the wrist was unsupported during measurements, the arm was evaluated as a whole. The study demonstrated that this haptic interface was suitable for the Upper Extremity (UE) assessment for persons with neurological impairments. The authors further concluded that the results were objective and repeatable. [10].

In our research, we use a semi-immersive workbench with force feedback provided by a haptic device (yielding sensory feedback) to develop a precise quantitative kinematic assessment tool and a training device for hand movement in healthy subjects and in victims with neurological impairments, especially for stroke patients [11,12].

To date very little has been reported on normal performance in VR environments concerning arm function. The aims of the present study are 1) to investigate whether any learning effects were achieved by repeating tests and 2) to develop normative data on 3-dimensional hand trajectories in a virtual environment for healthy subjects.

## Methods

### Subjects

The study included 58 healthy adults (right-hand dominant), 30 females and 28 males, mainly hospital or university employees. We sought persons who were novel VR users, i.e. did not work with VR equipment. The controls were recruited via direct contact, person to person, by telephone or by mail, or via their work manager. The age of the subjects ranged from 20 to 69 years with a mean of 42.8 years. Inclusion criteria were: no history of brain dysfunction according to history, no psychiatric illness or substance abuse, no dyslexia, Swedish as first language, no serious visual (including colour blindness and squinting) or hearing impairment, no acute illness and right hand dominant.

All subjects underwent a neuropsychological examination with the Barrow Neurological Institute Screen for Higher Cerebral function (BNIS) to confirm normal cognitive function. The BNIS [13] is a short screening test developed to systematically assess a variety of higher cerebral functions. It examines: language functions, orientation to person, place, and time; learning and memory skills; visual object recognition; right-left orientation; concentration; visual scanning and the presence or absence of hemi-inattention; the capacity to detect and manipulate information sequentially, constructional praxis; pattern recognition, affect expression, perception and control, and awareness of memory impairment.

All gave their written informed consent to participate and the study was approved by the Ethics Committee at Göteborg University (S549-03).

### Instrumentation

The VR environment consists of a semi-immersive workbench in which a stereo display and haptic feedback technology are combined into a form in which the user looks and reaches into a virtual space. A haptic device gives the impression of sensation feedback to the users when touching virtual objects. This gives the user the ability to interact with objects by touching, and moving their hand. A precise and detailed recording of hand movements is therefore possible. The PHANTOM^® ^Desktop™ haptic device  is a desk mounted robot sampling at 1000 Hz with 6 degrees of freedom. Here, we resampled the haptic x, y, and z data at 47–52 Hz. In this instance, the force feedback workspace was ~ 160 W × 120 H × 120 D mm.

### Procedure

We administered an arm test developed in a previous study [12]. The subjects had to move the haptic stylus to different targets (#32) in the virtual world generated by the computer. The targets appeared one after the other and disappeared when pointed at. Each target consists of a whole circle (diameter ~ 3.0° viewing angle). The target placements (#32) in the 3D space were apparently random to the subjects but were actually set according to a pre-set kinematic scheme for evaluation purposes. All tests were time stamped, giving the basic pattern of hand movement. The subjects were tested in three sessions within one week; each session consisted of three trials with two different handgrips. Two types of handgrip postures were studied, i.e. pen grip and cylinder grip. In this study a pen grip means that the haptic stylus is surrounded by the thumb, index and middle finger. A cylinder grip means that the haptic stylus is held in the palm, with the thumb against the four fingers. The procedure was standardized concerning sitting position and instructions in each test. The subjects were seated comfortably on a chair without an armrest, and both forearms rested in a neutral position on the table working with the arm unsupported. They were then instructed to pick up the haptic stylus first with a pen grip; this test was repeated three times. They were subsequently tested with the cylinder grip, and this test was also repeated three times. A 30 second rest between tests was allowed to reduce any possible fatigue effect. When the haptic stylus was picked up, a target became visible on the computer screen. The test started when the first target was pointed at. Each subject was asked to move as accurately and quickly as possible to each target. The assessment started as soon as the subject pointed at the first target.

All participants were tested between 10 AM and 4 PM. All tests were performed with the right hand.

### Data analysis

#### Kinematic data sampling and information processing

Hand position data (haptic stylus end-point) were gathered during each trial. The x-, y- and z-coordinates, which were time stamped, gave the basic pattern of hand movement. Time and distance to complete the whole exercise were also recorded, as this velocity was calculated. Movement quality was computed from the distance value. This is the distance traversed by the haptic stylus, calculating the length of the pathway divided by the straight line distance required to obtain a hand path ratio (HPR). Thus, a hand trajectory that followed a straight line pathway to the target would have an HPR equal to 1, whereas a hand trajectory that travelled twice as far as needed would have a HPR of 2.

Subsequently, the 3D kinematics of hand movement was visualized for one selected identical target-to-target movement for all subjects. In this case the midpoint trajectory of the trial was chosen, i.e. moving the haptic stylus from the one target to the next target. It should be emphasized that each subject generates approximately 288 (3 × 32 × 3) target-to-target movements through the entire dataset for each handgrip. This movement reflects a reaching movement (diagonally upwards, forward) in the physical environment.

For kinematical analysis of the target-to-target movement, the following were calculated: (1) time, (2) HPR, (3) max velocity (m/s) and (4) max acceleration (m/s^2^). In this case we used the second and third trials in the first test session.

### Statistical analysis

#### Test-retest consistency

The consistency between test and retest was evaluated with the 95% limits of agreement (LOA) method [14,15]. In this case we used the second and third trials in the first and the third test sessions (this method calculated the limits within which we expected the differences between two measurements by the same method to lie). To assess possible learning effects we used the Wilcoxon signed-rank test for paired scores between test sessions 1 and 3.

#### Normative data

We used the second and third trials in the first test session to establish normative data. Descriptive statistics, i.e. mean, standard deviations, median and 2.5-10-25-75-90-97.5 percentiles for the whole exercise and for the specific target-to-target movement, were calculated.

## Results

### Younger vs. older subjects

We examined the performance of the subjects by dividing them into two different age groups, i.e. younger adults (20–-44 years) and older adults (45–69). There were no significant differences in measures between the two groups for the whole exercise, and we decided to treat the material as a single age group.

### Test-retest consistency

The mean differences between the test-retest, SD of difference and 95 % limits of agreement (LOA) were calculated for the selected variables, shown in Table 1 (session 1). The Bland and Altman plots for the different parameters illustrating the test-retest agreement for both handgrips are shown in Figure 3. The assumptions of LOA were compared against the average of two measurements. The differences did not vary in any systematic way in both assessments and the two different grip types. All measurements were within the 95% limits of agreement. The analysis between session 1 and session 3 indicated a learning effect. The Wilcoxon signed-rank test for paired scores revealed that session difference was significant for all tested variables, p < 0.01 (Table 2). We then again tested for test-retest stability but this time within the second and third trials in the third test session (Table 1, session 3). The results also showed here no large variation in the two different grip types. All measurements were within the 95% limits of agreement.

**Table 1:** Test-retest consistency for sessions 1 and 3 for the cylinder and pen grips (n = 58). The mean differences between test and retest and 95% limits of agreement (LOA) for Time, Hand Path Ratio (HPR) and Velocity are given.

		Cylinder grip			Pen grip		
		
		Mean difference*	95% LOA		Mean difference*	95% LOA	
Session 1	Time (s)	2.14	- 8.83	+13.10	1.92	- 6.14	+ 9.99
	HPR	0.07	- 0.35	+ 0.49	0.07	- 0.26	+0.40
	Velocity (m/s)	0.00	- 0.06	+0.06	- 0.01	-0.04	+ 0.04

Session 3	Time (s)	1.22	-6.20	+ 9.74	1.22	- 4.24	+ 6.68
	HPR	0.02	- 0.32	+ 0.41	0.02	- 0.39	+ 0.42
	Velocity (m/s)	-0.01	- 0.08	+ 0.06	-0.01	- 0.07	+ 0.05

* Difference between subtests 2 and 3

**Table 2:** Changes in mean between tests 1 and 3 for Time (s), Hand Path Ratio (HPR) and Velocity (m/s) for the cylinder and pen grip.

	Cylinder grip			Pen grip		
	
	Session 1 Mean (SD)	Session 3 Mean (SD)	p value	Session 1 Mean (SD)	Session 3 Mean (SD)	p value
Time (s)	34.95 (8.59)	28,78 (6,07)	0.0001	37,49 (9.62)	30,14 (7,92)	0.001
HPR	1.77 (0.35)	1,69 (0.27)	0.001	1.86 (0.45)	1,77 (0.33)	0.01
Velocity (m/s)	0.25 (0.08)	0.29 (0.08)	0.0001	0.25 (0.07)	0.29 (0.09)	0.001

Table 3 give the mean (SD), median and percentiles (2.5-10-25-75-90-97.5) for time (s), HPR and velocity (m/s) for the cylinder and pen grips. Time (s), p = 0.01 increased with the pen grip as compared to the cylinder grip. In contrast, velocity (m/s), p = 0.03 and HPR, p = 0.18, did not have any significant effect on the difference in holding the haptic handheld stylus.

**Table 3:** Percentiles for Time (s), Hand Path Ratio and Velocity (m/s) for Cylinder and Pen (whole exercise).

		Cylinder grip			Pen grip		
		
		Time (s)	HPR	Velocity (m/s)	Time (s)	HPR	Velocity (m/s)
Mean (SD)		34.95 (8.59)	1.77 (0.35)	0.25 (0.08)	37,49 (9.62)	1.86 (0.45)	0.25 (0.07)

Median		33.1	1.66	0.24	35.6	1.73	0.24

	2,5	23.0	1.40	0.12	24.6	1.39	0.12
	10	26.6	1.42	0.16	29.0	1.48	0.16
Percentiles	25	29.4	1.54	0.20	30.7	1.60	0.19
	75	39,4	1,94	0.30	42.3	2.00	0.28
	90	45,8	2,42	0.38	45.1	2.28	0.35
	97.5	71,6	2,97	0.45	74.1	3.61	0.44

### Detailed recording of hand movements

The visual inspection of the detailed x-, y-, z-graphs for the hand trajectories for one target-to-target movement revealed a greater variability in movement pattern for the cylinder grip as compared to the pen grip. Data from ten "typical" subjects (5 females and 5 males) are presented in Figure 5.

The mean, median and percentiles (2.5-10-25-75-90-97.5) for movement durations, max velocity and max acceleration for the cylinder and pen grips are shown in Table 4. There were no differences between the cylinder grip and pen grip regarding time (s), HPR, max velocity (m/s) and max acceleration (m/s^2^), p > 0.01.

**Table 4:** Percentiles for Time (s), Hand Path Ratio, and Max Velocity (m/s) and Max acceleration (m/s^2^), for cylinder- and pen grip (target-to-target).

		Cylinder grip				Pen grip			
		
		Time (s)	HPR	Max Vel (m/s)	Max Acc (m/s^2^)	Time (s)	HPR	Max Vel (m/s)	Max Acc (m/s^2^)
Mean (SD)		0.99 (0.41)	0.72 (0.16)	0.54 (0.19)	0.17 (0.13)	1.05 (0.44)	0.71 (0.16)	0.52 (0.17)	0.16 (0.11)

Median		0.90	0.78	0.51	0.12	0.93	0.76	0.48	0.13

	2,5	0.46	0.28	0.28	0.05	0.47	0.31	0.28	0.06
	10	0.57	0.44	0.34	0.07	0.70	0.43	0.35	0.07
Percentiles	25	0.70	0.66	0.43	0.08	0.80	0.63	0.41	0.09
	75	1.13	0.84	0.60	0.19	1.13	0.82	0.60	0.17
	90	1.54	0.87	0.73	0.33	1.61	0.87	0.73	0.31
	97.5	2,33	0.91	1.08	0.65	2.36	0.91	1.00	0.51

## Discussion

The purpose of this study was to describe a novel technique for hand movement patterns analysis. The advantages of the proposed system are that it has the potential to take a single measurement that takes less than one minute and produce kinematic data. Further, the measures are objective and repeatable and provide quantitative data [10].

The results of this study indicate good test-retest reliability of the assessment. The use of multiple trials was recommended by Mathiowetz et al. [16] to improve test-retest reliability. The difference between sessions 1 and 3 does suggest a possible learning effect, which we consider to be advantageous. However, this effect is desirable when patients are training, and this information thus identifies the importance of having normative data to compare with.

A standardized test was developed for two different grip types. The cylinder grip was chosen so that the required movement would replicate the natural-world action of holding a handle. Secondly, many stroke victims' fine motor control with the hand and fingers is often impaired in the chronic stage of their disease [17], and the cylinder grip is then easier to use. The pen grip was chosen for the reason that it is a precision grip and enables the person to carry out a wide range of movements when using tools [18].

The data presented for the whole exercise on time, HPR and velocity showed no differences between the different grip types. This can be explained by the fact that a homogeneous group of subjects was investigated here, reducing the inter subject variability and thereby improving reliability measures. The x-, y-, z-graphs from the target-to-target movement in the different grip types were diverse. It seems that the hand path trajectories with the cylinder grip were more distributed, i.e. more dispersed within the workspace, than the pen grip movements, which were more arched and concentrated. When the subjects used the pen grip, the hand trajectories were more arched; this was not shown in the HPR measure, where the difference between the two grips was not significant. The velocity vs. time and the acceleration vs. time graphs indicate the possibility of saccade-like patterns of movement, with great inter-individual variability, but no clear difference was observed between subjects or grip types.

Evaluating the effects of therapy for rehabilitation practice is important both for rehabilitation personal and patients. Characterizing the features of reaching and quantifying specific variables allows therapists to treat specific deficits [19].

The normative data collected in this study will be used in a clinical evaluation unit (database), which will allow rehabilitation staff to measure and monitor patients' performance during assessment runs. All assessments will, by default, generate time-stamped motion data (x, y, z, yaw, pitch, roll and target press information) at 1000 Hz. These data are stored together with time/date and subject information for subsequent analysis.

## Conclusion

A new test was developed for UE performance in a virtual environment. The study demonstrates that it is feasible to collect a 3D quantitative kinematic measure in real time. Furthermore, these data can be stored in a database. Utilizing this system, the values of the progress in the exercises can easily be stored and re-accessed for further examination and evaluation.

## Competing interests

The author(s) declare that they have no competing interests.

## Authors' contributions

JB carried out the study, drafted the manuscript and made the statistical analyses. KSS and MR participated in its design and co-ordination and helped to draft the manuscript and make the statistical analyses.

**Figure 1:**
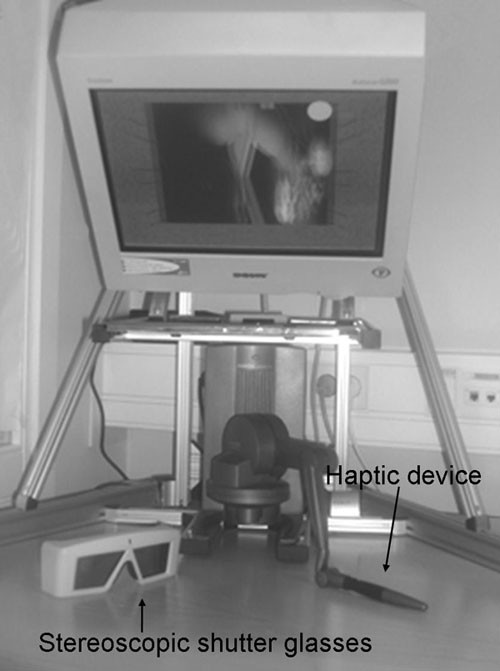
Semi – immersive workbench , with haptic device and stereoscopic shutter glasses.

**Figure 2:**
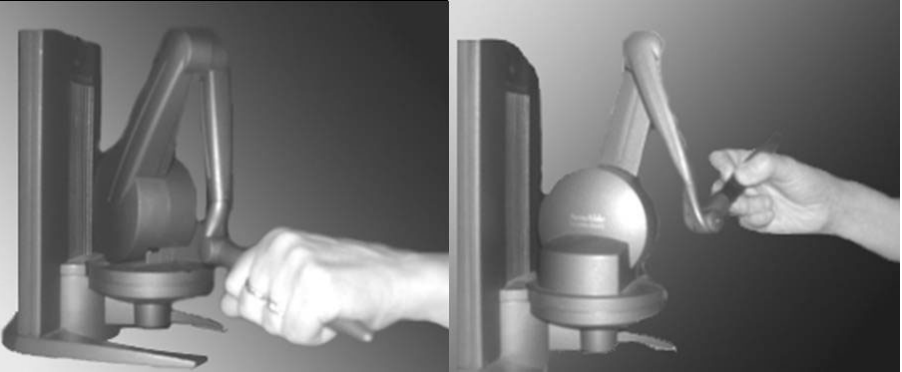
Different handgrip postures, cylinder grip (left) and pen grip (right).

**Figure 3:**
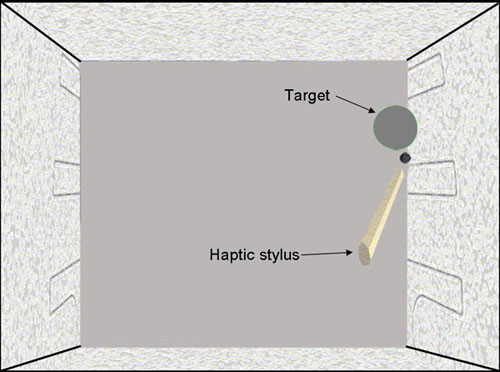
A screenshot of the stimuli.

**Figure 4:**
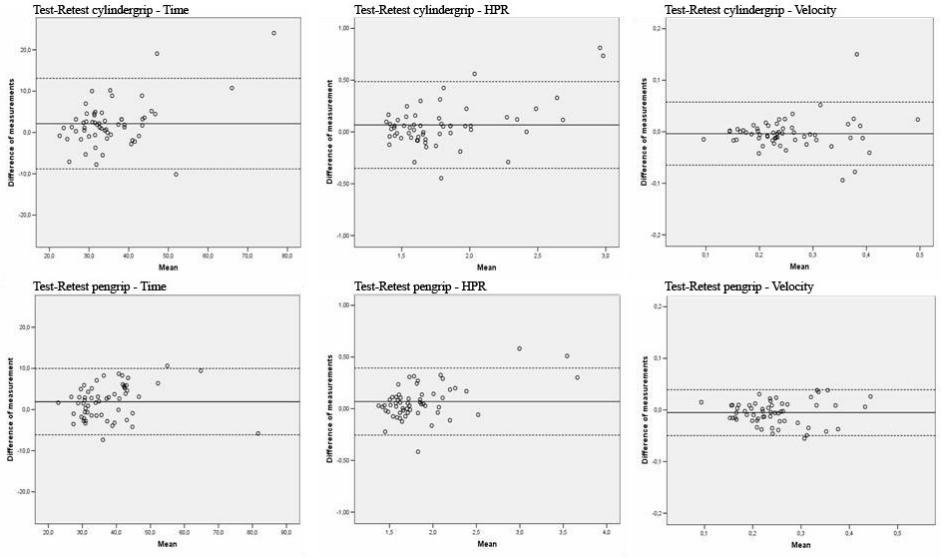
Scatter-plot of the difference between the second and third measure for Time, Hand Path Ratio (HPR) and Velocity within the first test session (n = 58) for cylinder and pen grip. The horizontal lines indicate the mean difference (middle) and the upper and lower limits of agreement.

**Figure 5:**
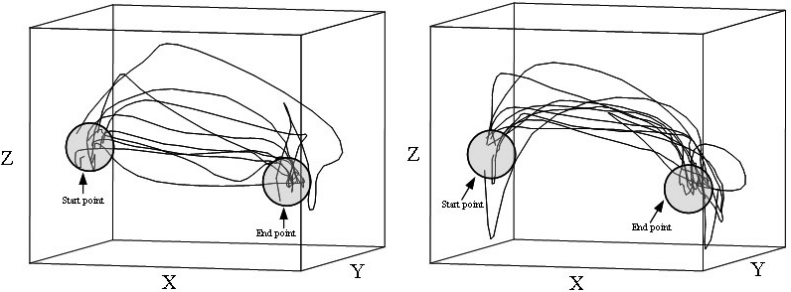
Detailed x-, y-, z-plot for the hand trajectories of ten subjects for one button to button movement. Left figure cylinder grip and right figure pen grip.
